# Reshaping the energy landscape of Crete through renewable energy valleys

**DOI:** 10.1038/s41598-024-57471-7

**Published:** 2024-04-05

**Authors:** Panagiotis Skaloumpakas, Elissaios Sarmas, Michalis Rachmanidis, Vangelis Marinakis

**Affiliations:** Decision Support Systems Laboratory, School of Electrical and Computer Engineering, National Technical University of Athens, Athens, Greece

**Keywords:** Energy management, Energy and behaviour, Environmental impact

## Abstract

Renewable energy valleys (REVs) represent a transformative concept poised to reshape global energy landscapes. These comprehensive ecosystems transition regions from conventional energy sources to sustainable, self-reliant hubs for renewable energy generation, distribution, and consumption. At their core, REVs integrate advanced information and communication technology (ICT), interoperable digital solutions, social innovation processes, and economically viable business models. They offer a vision of decentralized, low-carbon landscapes accessible to all, capable of meeting local energy demands year-round by harnessing multiple renewable energy sources (RES) and leveraging energy storage technologies. This paper provides an overview of the key components and objectives of REVs, including digital integration through advanced ICT technologies and open digital solutions that enable the seamless management of RES within the REV. The social innovation aspect via the REV’s active communities is also examined, encouraging their participation in the co-design, implementation, and benefit-sharing of renewable energy solutions. In addition, business viability through sustainable business models central to the REV framework is proposed, ensuring affordability and accessibility to all stakeholders. The paper presents a case study of Crete, showcasing how the REV idea can work in real life. Crete utilizes various energy sources to become energy-independent, lower carbon emissions, and enhance system resilience. Advanced energy storage technologies are employed to ensure supply and demand balance within the REV. Situated on the picturesque island of Crete, Greece, it is pioneering the establishment of a Renewable Energy Valley ‘Living Lab’ (REV-Lab), integrating Community Energy Labs (CELs) as innovation hubs. This initiative exemplifies the REV model, striving to create a digitalized, distributed, and low-carbon landscape accessible to all residents throughout the year.

## Introduction

Today, the European energy sector is facing unprecedented changes. In less than 4 years, amidst countries’ efforts to decarbonize and shape a more inclusive energy transition, Europe has been challenged by two ‘once-in-a-lifetime’ shocks. The COVID-19 pandemic, leading to repeated shutdowns of large parts of the economy for extended periods of time, has added to this crisis. In addition, swiftly turning into a global geopolitical crisis, the 2022 Russian invasion of Ukraine gave rise to new energy troubles, notably the spikes in energy costs and the ever-highlighted need for energy independence. With these recent shocks and in anticipation of the energy transition challenges ahead, the European Commission (EC) presented the REPowerEU Plan^1^ to ramp up the production of green energy, diversify the current energy supplies, and reduce the use of fossil fuels. This plan further strengthens and complements the current EC’s high-profile policy initiatives and programmes for more affordable, secure and sustainable energy, such as the European Green Deal^2^ and the Fit for 55 package, the Digitalising of Energy Action Plan^3^ and the European Pillar of Social Rights Action Plan^4^. From a global perspective, the accelerating increase of population along with radical climate change consequences also call for radical changes of humanity’s energy consuming habits. The United Nations has declared the development of sustainable communities across the world as a main contributor to achieving five out of the seventeen Sustainable Development Goals for the 2030 Agenda for Sustainable Development^5^.

All these actions leverage the sustainable transformation and digitalization of the energy system infrastructure, where smart grids, demand response (DR) programs, and extended informatics enhance decentralization, interconnection, and grid-resilience, which are vital in enabling greater utilization and integration of RES, unleashing and exploiting the flexibility potential in RES-dominated energy systems. Aiming to upgrade the grid to a more versatile system, capable of rising to the challenges of sustainability, while simultaneously displaying greater performance and security, is a primary objective^6^. A robust social capital along with cooperation among active citizens are equally important in this endeavour, as they can both drive individual willingness towards sustainable communities as well as push for the development of energy policies. On the other hand, the formation of sustainable communities can also result in the enhancement of the quality of energy management as it has been shown that collective self-consumption results in significant economic benefits for the citizens^7^ . Energy citizenship^8^ can promote this ownership at the crossroads of fossil fuel divestment and transition justice. Incentives such as Renewable Energy Communities (RECs), bolstered by frameworks like the Recast Renewable Energy Directive (RED II), aim to decentralize and democratize the electrical energy market, enabling citizens to actively participate in community-based energy production, storage, and sharing^9^. By democratizing energy, decentralizing production, and establishing active energy communities, energy policies can have longer-term sustainability and leave no one behind.

The formation of sustainable energy communities hinges significantly on the interplay between social capital, energy efficiency measures, and policy frameworks. Research shows that social contexts, influenced by various dimensions of social capital such as structural, relational, and cognitive factors, play a crucial role in fostering community participation in energy initiatives. This involvement is facilitated through familial pathways and broader social channels, where higher social trust and neighbor interaction positively impact willingness to engage^10^. Despite academic discourse on energy decentralization and democratization, policy impact remains limited, with centralization prevailing at the national level. To get around this problem, authors argue that RECs should be recognized as legal entities within their socio-legal institutions. This would help with a fair transition to a new energy paradigm. Sustainable communities, comprising of collective formations of stakeholders that operate on a scale located somewhere between the individual and the local government, formed in a bottom-up approach where citizens participate in collaborative processes^11^. Drawing from experiences in Europe and beyond, there is growing support for this model, contingent upon purposeful legislative action and interdisciplinary collaboration. Such efforts aim to provide a coherent platform for realizing sustainable energy communities and advancing energy justice goals^12^.

However, it is crucial to acknowledge the inherent limitations of conventional energy community models. First of all, the reliance on a limited set of renewable sources within a specific geographic location may lead to sub-optimal energy production. Analysis of different energy community projects revealed the dominance of traditional self-consumption place-based communities, while business models involving differentiated services such as demand flexibility, aggregation, energy efficiency, and electric mobility are still scarce^13^. In addition, the focus of traditional energy community models on local solutions and small-scale renewable energy systems may not align with the operational priorities and requirements of Distribution System Operators (DSOs) and Transmission System Operators (TSOs), which remain heavy infrastructure actors and regulated entities that are dependent on the right framework conditions to deliver. The fast increase of RES deployed and the growing role of new active participants in the European energy market, like energy communities, additionally aggravates the conditions. This exclusion hinders seamless integration into the larger energy landscape and makes access to the grid still fragmented and scattered^14^. As a result, traditional energy communities may struggle with scalability issues, particularly in meeting the energy demands of larger regions or populations due to their small-scale renewable energy systems. The focus on local solutions, while commendable, may inadvertently overlook the synergies that can arise from interconnecting diverse energy communities on a broader scale through collaboration with grid operators. To enhance grid operators’ involvement in energy community models, measures such as developing frameworks for collaboration, establishing regulatory incentives for grid integration, and implementing standardized procedures for capacity allocation and access are essential.

In this respect, there is a push for innovative, viable and efficient solutions, such as REVs, namely decentralized renewable energy systems that enhance energy autonomy, ensure a more secure supply, and reduce total energy expenditures while maintaining stability on a broader scale. REVs provide a potent response to this call with focus on combining innovative electrical energy communities, next generation heating and cooling networks^15^ as well as modernised transportation infrastructure, covering a huge rate of energy needs for large areas and populations^16^. REVs aim to interconnect distant energy communities, each one producing energy from the ideal RES depending on its geographic location, and implement a greater variety of renewables such as geothermal, biomass, biogas/bio-methane, along with hydrogen, combined with battery energy storage systems. In order to ensure the sustainability and autonomy of the whole system, minimal environmental footprint of the grid, and low cost of energy for the citizens, a plethora of state-of-the art technological tools will be utilized, namely, business models, the exploitation of the abundance of available data from smart metering Internet of Things (IoT) equipment, as well as the evolution of Artificial Intelligence (AI) which has reached unprecedented levels within the last decade^17,18^.

## Hindrances in REV implementation

**Figure 1:**
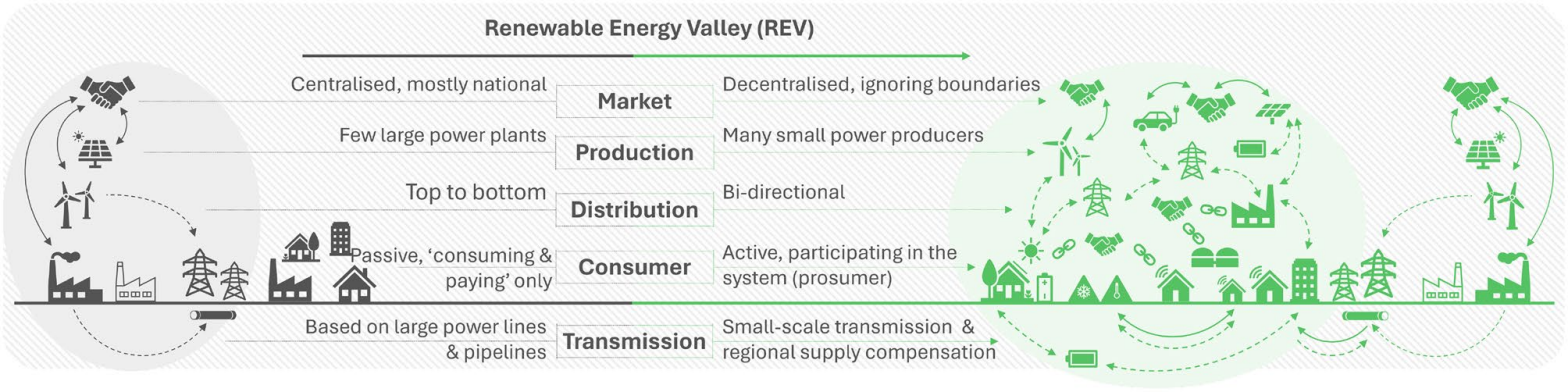
The transformed energy landscape through the prism of REV implementation.

In navigating the evolving energy landscape, the realization of REVs demands a profound transformation across social, technological, and business dimensions. As technological innovations align with societal needs, the current energy sector faces a pivotal juncture requiring fundamental shifts. This section explores the intricate interplay of social practices, technology adoption, and business models in the context of REVs, highlighting existing challenges and barriers that impede their scalable deployment. From reshaping energy markets to fostering consumer proactiveness, addressing these multifaceted challenges is essential for propelling REVs into a sustainable and decentralized future (Fig. 1).

### Current status of the energy landscape

Great energy transitions occur when innovations and technological developments align with society’s needs^19^. While figuring out the correct path to establishing a future decarbonised and decentralised energy grid, fundamental modifications should take place to create fruitful soil for new technologies. Reforming the foundations of the five pillars of the energy sector is necessary for REVs to manifest their capabilities.

Regarding the first pillar, the energy market, as of its structure it requires changes to support the founding and the proper functioning of decentralized endeavours. As of now, in every centralized market, the day-to-day procedure involves producers submitting detailed cost-related information to the Electricity System Operation (ESO) and the latter deciding how much each generation unit should produce^20^. On the contrary, heading towards a decentralised system, Peer-to-Peer (P2P) programmes are widely discussed towards finding ways of power balance and stability in the network, as it allows direct energy sharing among consumers and producers within the local grids^21^.

Achieving the formation of local grids able to replicate large-scale markets with P2P energy trading, requires the further use of Distributed Renewable Energy Sources (DRES). The production of electrical energy, considered to be the second pillar of the energy sector, possesses an immutable form producing a great extend of the global energy in a centralised way. Moving away from the latter requires a combination of RES all available in residential, community and utility scales. Both grid interactive and grid non-interactive microgrids able to produce enough energy to form robust, decarbonised and sustainable systems^22^.

By the increasing DRES merit in the system, DSO’s role should be adjusted in hope of aligning with the new needs of the market. The distribution system currently plays a critical role in the efficient, reliable, and safe delivery of electricity from power generation sources to end-users, the so-called top to bottom system. Its unidirectional shape should reshape into a bidirectional operator able to support the needs of microgrids. Each microgrid produces and consumes energy locally, participating in energy trading market of numerous microgrids which exchange power depending on their needs. A modernised DSO should be able to supervise the power flow control and market operation of interconnected microgrids^23^. The essence of microgrids and energy communities is to intake power when needed and offer power when achieving a surplus. Building distribution systems that can optimise these operations between decentralized microgrids can result in robust and resilient power systems^24^. This could entail developing new market structures, such as P2P energy trading platforms, to facilitate transactions between interconnected microgrids. Additionally, specific requirements for microgrid operation and integration, such as technical standards for grid connectivity, grid resilience, and power flow control, may need to be established to ensure the safe and efficient functioning of the distribution system in the context of a more decentralized energy landscape.

Establishing a new form of consumer identity constitutes an argument around the fourth pillar of the energy sector, the users of the system themselves. Up until now, the only choice that energy consumers have is the choice of energy supplier. But democratizing energy nurtures the notion of offering the ability to the consumer to participate in their community’s decisions^25^. With a correct mix of economic and non-economic factors could tempt consumers transitioning to prosumers. Low energy price by small investment in DRES, environmental impact by participating to a decarbonised energy grid and fostering a sense of community and cooperation by trading energy, pose as significant motives to transit from consumers to prosumers^26^.

The transmission system constitutes the fifth and last pillar of the energy sector that requires fundamental shifts in its processes in order for REVs to become implementable in a modern energy grid. It is usually operated by the TSO^27^, who has a plethora of jurisdictions, depending on the regulation of each country. While TSOs traditionally provide EU-wide interconnection of electricity systems to maintain frequency synchronization and system inertia, focusing on developing new power lines for the interconnection of decentralized microgrids offers benefits for all the involved parties^28^. This shift in focus allows for energy exchanges to occur as locally as possible, reducing energy losses associated with long transmission distances and promoting a more resilient and efficient energy grid. Therefore, while the TSO’s central role in maintaining system stability remains important, adapting to support the interconnection of decentralized microgrids is essential for optimizing the transmission infrastructure and facilitating the implementation of REVs within a modern energy grid.

### Existing limitations in social, technological, and business aspects

Even though new methods emerge from the requirements of voids in every science, obstacles exist in the current status quo that refrain them from flourishing. In the case of REVs, the current social practices, technology adoption and organizational and business models are not sufficiently mature to enable them scaling up and replicating. Challenges and barriers exist, that are preventing the full deployment of local or regional renewable energy system solutions for electricity, heat and fuel needs in the following three dimensions.

First and foremost, there is the social aspect of the implementation challenges and in particular the willingness and readiness of the people to undertake their new role and become the centre of the energy system. While energy may be ubiquitous, the mechanisms delivering it in various forms are complex and rely on a network of science, engineering, economic market and public regulation. This network is essential for satisfying demand and maintaining physical structures and supply capability. Consumers primarily see price signals, but not the connection between sources of energy (fuels) and the technology that delivers energy or the direct benefits of either conservation or the connection of energy to shifts in climate. Understanding tariffs, grid dynamics and technology options can be discouraging for non-experts. The lack of informative incentives, aiming to tackle energy illiteracy, strengthens the comfort barriers and fears of citizens about decentralization.In order to transition to a more human centric system citizens need to understand how the energy market works, how they can be part of it, and how they can benefit from their choices. However, shifting consumer behaviour is complex, as deep-rooted habits and biases hinder energy-efficient choices and DR participation^29^. Feasibility, affordability, trust, policies, market access, and knowledge gaps all have an impact on adoption, while consumers frequently lack the information they need to make informed decisions and have faith in service providers, regulators, and technological matters. More actions should be taken in the direction of informing people about the financial motives at stake, the energy independence and environmental impact of participating in a REV project. For a successful and long-lasting REV, trust should be built among the members.

Regarding the technological dimension, a void exists in the digital tools arsenal for setting up, developing and replicating REVs. Planning large scale projects demands the interaction of many parties of different expertise, meaning that most of them would need to utilize innovative digital tools to convey their systems status and possible limitations. At the same time, most non-community members have lesser knowledge about the total operation of REVs. Implementing digital tools that will aid the planning and tracking of their status is considered a crucial step for developing an attractive framework^30^. Apart from tools offering faster and easier to understand services to consumers, protocols must be raised to protect the latters’ personal data. Consumers raise huge concerns about their personal information data sharing. Developing a business model that secures the operation of the REV demands smart meters transmitting the users’ electricity usage on a real time level. With no protocols available for sensitive data protection, consumers and prosumers become vulnerable to malicious data mining attacks, exposing their living habits^31^.

From a business standpoint, the industry is lacking in providing models based on sharing economy and social innovation. At the moment various conceptual models are being tested around the globe, on innovative ways to modernize the energy market^32–34^. But still the political and regulatory environment must develop frameworks, guaranteeing the necessary conditions for citizens to transition to a more prosumer centric system and in concurrence with the undergoing electricity market design reform^35^.

## Methodological framework for REV implementation

Addressing the above-mentioned hindrances, it is imperative to articulate the reciprocal interaction among social, technological, and organisational/business layers to enable the effective engagement of consumers at the individual and/or REV-level. For a successful implementation of REVs, a social, technological, and business framework is crucial to be deployed, aimed at aggregating multiple renewable energy carriers and promoting consumer activation in order to increase local and system-level energy efficiency while at the same time aiming to increase local security of supply. The directions in each layer to enable the REV implementation are detailed below, presenting the proposed broad methodology framework.

- In the social layer, a combination of qualitative and quantitative research driven by Social Sciences and Humanities (SSH) aspects will be leveraged to bring together the energy value chain and citizens (consumers and prosumers), supporting them throughout the process of co-designing and co-creating REVs. Attention and resources should be directed towards boosting the energy literacy levels of citizens, as their participation plays a crucial role in the expansion of REVs role in society. Insights from social and behavioural science will be utilized to identify the needs and values of different types of citizens, embedding their technical, social, economic and institutional perspectives, and design effective actions that encourage consumers to take ownership of local energy projects and participate in the energy markets. Tools designed specifically for raising awareness on how they can contribute to the decarbonisation of the energy system while simultaneously they can pursue their individual and collective economic^36^, environmental and social goals^37^. Moreover, public awareness campaigns, and educational activities will demonstrate the benefits of RES integration, storage, and energy market participation in relatable terms to leverage social innovation and improve energy literacy, empowering the “Leave No One Behind” principle. Energy literacy initiatives can have an astounding impact on bringing citizens together towards common goals and assist them in taking full control of their energy practices and behaviours at the individual and REV-level^38^.
- The technological layer will be encompassed by state-of-the-art digital solutions focusing on interconnecting remote communities, targeting to fully cover their energy needs with their own energy production, while securing the regular operation of the system, safety and benefits of members and maximizing the potential of RES. The backbone of this layer should be in the form of a Digital Twin (DT) supporting the local energy grid and consumer services. business model is a simulation process that integrates multiple disciplines, physical quantities, scales and probabilities^39^. With the emergence of smart sensors, 5G communications, Artificial Intelligence and Big Data analysis, it has become easier to develop virtual copies of constructions of interest. In Power Systems in particular, they can be found under the terminology Power Systems Digital Twins (PSDT)^40^. A PSDT is essential for the operation of REVs, as the virtual copy of the physical system can manage the large amount of data deriving from every sensor in the system, performing various operations at the same time. Such operations would assist the stakeholders in efficiently coordinating, managing and operating local energy grids, as well as provide data-driven services to individual consumers for energy efficiency and load management^41^. A robust energy power system should carry a plethora of tools that guarantee the regular and efficient operation of the system as well as the prediction and prevention of possible faults and inadequacies^42,43^. The efficient coordination of the local energy grids will involve utilizing power flow simulations to model the behaviour of the grid under different values for the parameters of loads, generators, and weather patterns. The ability to optimize the operation of the system under different case scenarios that have already been studied by the DT’s functions will stand out as a key factor to the long-term performance, minimal environmental impact and high energy autonomy of the REV.
- The organisational/business layer will include the design and validation of new market roles, as well as cross-value chain/cross-energy carrier and hybrid private/public sharing economy business models. Business model innovation is deemed crucial for the development of REVs as the commercial viability of innovative technologies is achieved through sustainable business models^44^. With the establishment of REVs, new roles are being formed that can be covered by various entities. According to the Council of European Energy Regulators^45^ three business models are categorised for collective services: community owned generation assets, virtual sharing over the grid and sharing of local production through community grids. REVs’ communities are being portrayed by the virtual sharing over the grid category where the public electricity grid is being utilised to share the renewable energy produced among its members. The excess or deficit of electrical power is carried out by the energy supplier, who is also responsible for organising the sharing. Apart from that, citizens are allowed to buy equity and gain access to project governance over renewable energy projects. Municipalities will undertake a leading role in the operation of REVs as their involvement in the market as municipal energy companies could promote energy decentralisation in business activities^46^.

## Proposed demonstration site: the case of Crete

### Background

The main objective is to create a REV-Lab in the Crete island, combining leading-edge ICTs technologies^47–49^ with social innovation processes of co-design and co-creation by involving stakeholders (including citizens and businesses) and easy-to-adopt efficient business models. Crete is the fifth largest island in the Mediterranean Sea, regarding both its area and population. Given its intensive land morphology, Crete constitutes a mosaic of discrete geographical entities with considerably different climate conditions and, consequently, energy needs. At the same time, Crete combines high wind potential, particularly in the mountain ridges, and high solar radiation, as typically also happens for all Aegean Sea islands. Crete, precisely thanks to its large size, also has significantly high biomass resources, coming from agricultural processes, stock farming and urban organic wastes^50^. It has been estimated that with the exploitation of these biomass resources, annual heat 2,4 times higher than the currently annually supplied heat from diesel oil for indoor space heating in Crete, can be produced^51^. The vicinity of all coastal settlements to the sea, where also the main portion of the touristic activities are implemented with corresponding increased energy needs, particularly for indoor space cooling, creates favourable conditions for the installation of open-loop geo-exchange plants, specifically for the coverage of this essential final energy need. Thus, the effective and rational energy transition in Crete, aiming at near full energy independency, should be based on the development of a cluster of community energy projects, powered with multiple renewable energy carriers. Given its size, the extensive energy demand and the different conditions met in coastal, mountainous and plain regions, Crete Island features as an ideal field for implementing pioneering energy transition initiatives, such REVs. The active participation of local communities in the implementation of the energy transition projects, through the involvement of citizens, organised under energy communities, and stakeholders (local authorities, SMEs, etc.) constitute an important objective towards the achievement of energy independency and energy democracy. These are the main objectives of existing energy communities: the implementation of all available alternative technologies for covering electricity, heat and fuel needs in Crete, with the active involvement of the local communities, for a fair and effective energy transition for all.

**Figure 2:**
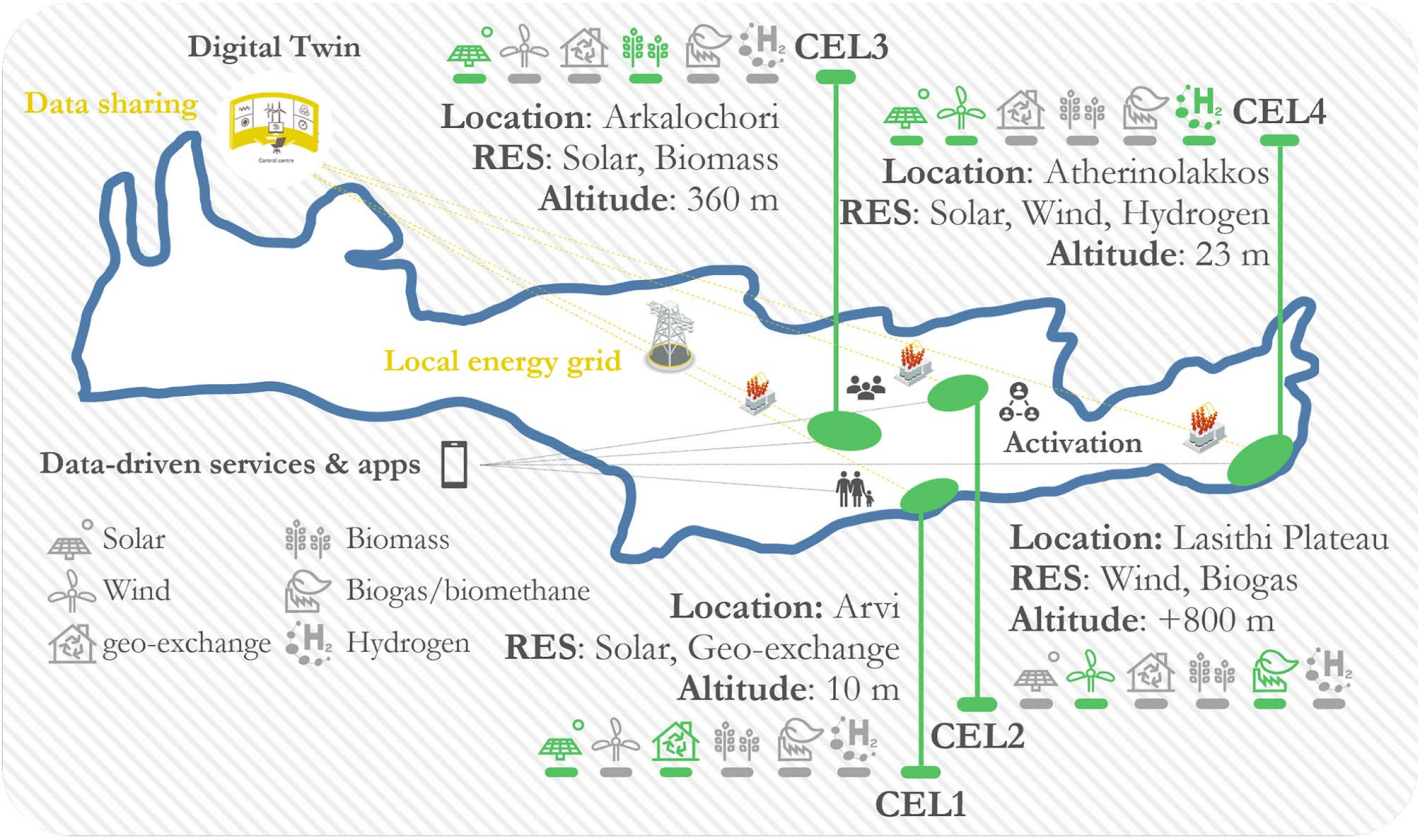
Overview of the geographical location of the CELs that will support the demonstration activities of the REV in Crete.

Focus will be on the transition from a centralised carbon driven scenario to a distributed, renewable, low carbon landscape, driven by the Crete REV-Lab, fully covering the local energy needs on an annual basis by multiple renewable energy carriers for electricity, heat and fuels (solar, wind, geothermal, biomass, biogas/biomethane, hydrogen) and leveraging energy storage technologies. The Crete REV-Lab will be made up of four Community Energy Labs (CELs) located in distinct sites across the REV, conceived as Innovation Hubs (Fig. 2). The CELs are adequate to demonstrate in real-life conditions an improved data-driven multiple carrier grid management and operation for sustainable and cost-effective production and storage of renewable energy to fully cover the local energy needs of the REV-Lab (22.840 MWh/y) on an annual basis (Table 1). A detailed description of the infrastructure and the conditions that led to the selection of these areas as potential CELs in Crete is provided below.

- **CEL1**. Arvi is located in the southern coast of Crete, in the eastern part of Prefecture of Heraklion. The settlement’s proximity to the seashore creates the prerequisites for the installation of an open-loop geo-exchange plant and a district cooling hydraulic network. The disposed heat from the conditioned indoor space during the summer can be used to produce domestic hot water through decentralized heat recovery systems. The same geo-exchange can be used for the coverage of the rather minor heating needs of the settlement during the winter. The proposed open-loop, geo-exchange plant in the coastal settlement of Arvi will lead to a substantial reduction in electricity consumption for indoor space heating and cooling compared to the currently used air-to-air heat pumps for the involved CEL members. Finally, the abundantly available solar potential can be utilised for electricity production with PVs, for the annual compensation of the electricity consumption by the involved users.
- **CEL2**. The Lasithi Plateau is located in the mainland of the Prefecture of Lasithi, in the middle of the Dikti Mountains. Due to its mountainous location, the area is characterized by a cold climate, and indoor space heating is required for the majority of the year. Given the existing agricultural and stock-farming activities, a biogas plant will be installed that will have a reception area for liquid and solid bio-based waste streams. The biogas produced from the plant will feed a combined heat and power (CHP) engine, eliminating the use of expensive diesel oil for indoor space heating. Additionally, 50 small windmills of 3 kW nominal electrical output can be installed in the area for electricity production, operating as a net-metering plant for the electricity consumption of the involved members. The electricity consumption of the involved members will be compensated with the production from the windmills, as in the case of the PV plants in Arvi.
- **CEL3**. Arkalochori is located in the central region of the Prefecture of Heraklion, exhibiting characteristics of a plain-semi-mountainous terrain. This geographical location results in significant requirements for both heating and cooling within the area. There exists a significant quantity of solid biomass resources, primarily derived from the pruning of olive trees. A biomass plant will be installed to utilize olive tree trimmings, providing a reliable and cost-effective source of fuel for local members. The boiler will have enough power to produce heated water to sufficiently cover the community members’ needs. This alternative heating option offers a significantly lower cost compared to the current method, which relies on the use of diesel oil. In close proximity to the town, it is feasible to establish a small wind turbine facility. Additionally, the installation of a small wind turbine facility and the integration of PV installations in close proximity to the town can further increase the electricity generation capacity. In conjunction with these, the implementation of decentralized storage facilities will result in the annual offsetting of electricity usage for end-users.
- **CEL 4**. In Atherinolakkos, an existing hydrogen production facility annually produces hydrogen using renewable electricity from dedicated PV plants and, in the future, from a large-scale near-shore wind turbine project. The hydrogen production contributes to energy flexibility, addresses seasonal energy demand surges, and reduces the island’s reliance on oil-dependent generators, consequently lowering GHG emissions. The strategic location enables cost-effective introduction of PV power into the electricity and hydrogen market. Increasing the hydrogen production facility’s capacity factor is critical for economical operation, achieved by extending full load hours, reducing investment costs, and lowering the levelized cost of hydrogen (LCOH). Expanding green hydrogen utilization will enhance energy security, cover mismatches in production and consumption, and support decarbonization in transport and power. The planned deployment of hydrogen fuel cell-electric buses will showcase the efficient use of hydrogen in mobility. An additional end-use demonstration of the hydrogen produced is its utilisation in fuel cells for combined heat and power production, covering the thermal and electricity needs of nearby communities.

**Table 1:** Local energy needs and planned local RES and storage based on the input from energy stakeholders that operate in Crete.

REV Lab	Local energy needs (MWh/y)	PV (MWh/y)	Wind (MWh/y)	Biomass (MWh/y)	Biogas (MWh/y)	Geothermal (MWh/y)	Batteries (MWh/y)	Hydrogen (MWh/y)
CEL 1	4.160	660	–	–	**3.500**		1.500	–
CEL 2	870	–	170	–	**700**	-		–
CEL 3	5.660	1200	260	**4.200**	700	–		–
CEL 4	12.150	–	–	–	–	–		**12.150**
Total	22.840	1.860	430	42.00	700	3.500	1.500	12.150

Values in bold indicate the main RES that will cover the energy demands for electricity and heating in each CEL.

In the endeavor to interconnect remote communities and reinforce them in meeting their energy needs through the REV in Crete, the integration of state-of-the-art methodologies becomes imperative. The implementation of tools for the proposed REV should address the social, technological, and business aspects as described in the suggested methodology framework.

### The social aspects of creating a REV

More specifically, the social layer will provide a Social Science Framework adopting a variety of established SSH methodologies to support the foundation and sustainability of the REV, through social systems that improve the participation of all parties involved. One of the initial steps is the creation and coordination of the REV-Lab and CELs in the Crete Island. Key consumer groups will be identified and characterised by utilising AI-based algorithms, along with a variety of criteria and characteristics, in order to achieve a better behavioural segmentation of consumers and, ultimately, to detect and semantically interpret the socioeconomic characteristics of each segment. Combining cross-sectoral fields of expertise will pave the way to structure a solid clustering methodology based on a complete set of social-economic, geographic, gender, socio-cultural and socio-political factors. By collecting and analyzing social information to understand the intricate links between energy, climate, and social dynamics throughout the operation of the REV in Crete will assist in acquiring knowledge for the successful replicability of REVs and explore how community behaviors and social structures influence energy choices and climate outcomes. This social information will inform the development of targeted and effective interventions. In addition, interaction between all parties involved is of utmost importance to develop a common understanding and social ties among the CELs set up in distinct sites within the REV-Lab. The target is to cultivate an open innovation ecosystem that encourages the exchange of ideas, knowledge, and best practices among diverse communities. This approach fosters a sense of shared purpose and collaboration. The application of social innovation methods and tools for the engagement of citizens and business-related actors, considering diversity and particular condition of each energy community within REV, will support the adoption of new practices and definitions of RES-based solutions at the technical-social interface. Lastly, community members as well as stakeholders are premeditated to be involved in financial and non-financial incentives aimed at developing services that satisfy the needs of consumer groups and encourage sustainable energy practices.

### Technology enablers for REV implementation in Crete

In the technological layer, a variety of simulation tools, collaborative models, and decision-support mechanisms for all operations involved will be combined. Initially, a REV readiness assessment toolkit will be implemented to support the operation of the REV in Crete. The toolkit, equipped with state-of-the-art models, will provide science-based insights into the future power system and its environment, aiming to help energy stakeholders identify optimal adaptation and resilience investments for a reliable energy grid. Furthermore, those models will possess the capability to assess the reliability of distribution systems and battery systems for grid support services by simulating the performance of such systems under different operation strategies. In addition, this toolkit will be a major contributor to the development of a new REV planning process.

Furthermore, an interactive decision support tool will adapt the proposed readiness assessment toolkit, AR applications, and existing computation modules for sizing electrical assets and optimizing heating and cooling assets and networks. The front end of the decision support tool will integrate Geographic information system (GIS) capabilities, guided questionnaires, and existing databases. The developed tool will allow users to assess the economic and social benefits of becoming an active member of a CEL, and assess different scenarios based on the elements of the case. The value chain energy stakeholders-oriented view will be able to assess the readiness and maturity of several consumers to be aggregated as an energy community against several different criteria.

Interoperability will be featured in all the processes of the REV functions, ensuring the ease of data exchange between the systems of the REV in Crete without diverging from existing regulations. For the optimal operation of the REV and the local energy grid, a semantic interoperability framework will be implemented. This framework will leverage advanced semantic interoperability enablers to drive the creation of digital services for the energy sector. The resulting interoperability enablers will comply with a set of standards for minimum interoperability, aligned with a Semantic Interoperability Framework (SIF) and the data space connector. This will enable seamless semantic integration of energy assets in the REV and facilitate P2P trading of renewable energy assets between energy communities. Energy Data Space-compliant tools will be developed to exploit the interconnectivity with local business platforms. These tools will explore potential data services that will facilitate the empowerment of consumers and will be supported by harmonised and extended Smart Grid Data Models, business DR standards, ontologies, and languages.

Another one of the prerequisites for the implementation of REV in Crete, is the data security of CEL’s participants and the regulatory compliance of operations between the different layers of the system. In this effort, protocols will be followed aimed at enabling arbitrary integration of field assets and legacy systems with cloud back-end and data analytic services, enhancing security over legacy protocols, and adopting standardised APIs to allow for system-level interoperability.

The use of cutting-edge technologies will ensure the REV’s operation in Crete is successful and sustainable. As networks become increasingly critical, the ability to operate as a microgrid or off-the-grid for a short period before re-synchronising with the wider network will become increasingly important. For this case, a robust simulation tool for microgrid-mode network operation and contingency analysis will be implemented. This tool will be extended to provide contingency and transient analysis capabilities for transmission and distribution operators. Dynamic Phasors^52^ will be utilised to analyse study cases related to critical operation at frequencies that differ from the standard frequency of operation in the grid. By stabilising the system and returning it to a safe state, this tool can lead to breakthrough possibilities for microgrid operation and off-the-grid contingencies, with high accuracy and using less computing power.

Moreover, system flexibility is of crucial importance for DRES-based systems, as in the case of the REV in Crete. In this context, an optimised scheduling tool for energy management will be utilised in the CEL to reckon their members’ behaviours, and create new business models with shared assets, with a special focus on the impact of storage systems’ degradation. The tool will be able to efficiently manage energy resources, optimise the utilisation of flexible assets, and reduce energy costs while promoting the use of RES. In alignment with the optimised scheduling tool, new models and approaches, like transfer learning and incremental learning, will be exploited to optimise the forecasting accuracy of the models^53^. The implementation of an energy forecasting module will enable the project to efficiently plan the energy infrastructure and maximise energy efficiency, helping to ensure that the REV becomes a sustainable, low-carbon region.

A system-of-systems DT for Virtual Power Plants (VPPs) will be developed, functioning as a connector for the above state-of-the-art technologies, and will be extended to cover the use case demonstrators in each CEL of the REV. By utilising the DT for community RES aggregation in distribution networks, short-term decisions can be made, both semi-automated and human-dependent^54^. In addition, the operations of the DT will support a real-time approach, augment the availability of data, and provide information on network parameters, such as congestion of lines and load on transformers, for the current and potential forecasted states of the suggested network configurations^55^.

**Figure 3:**
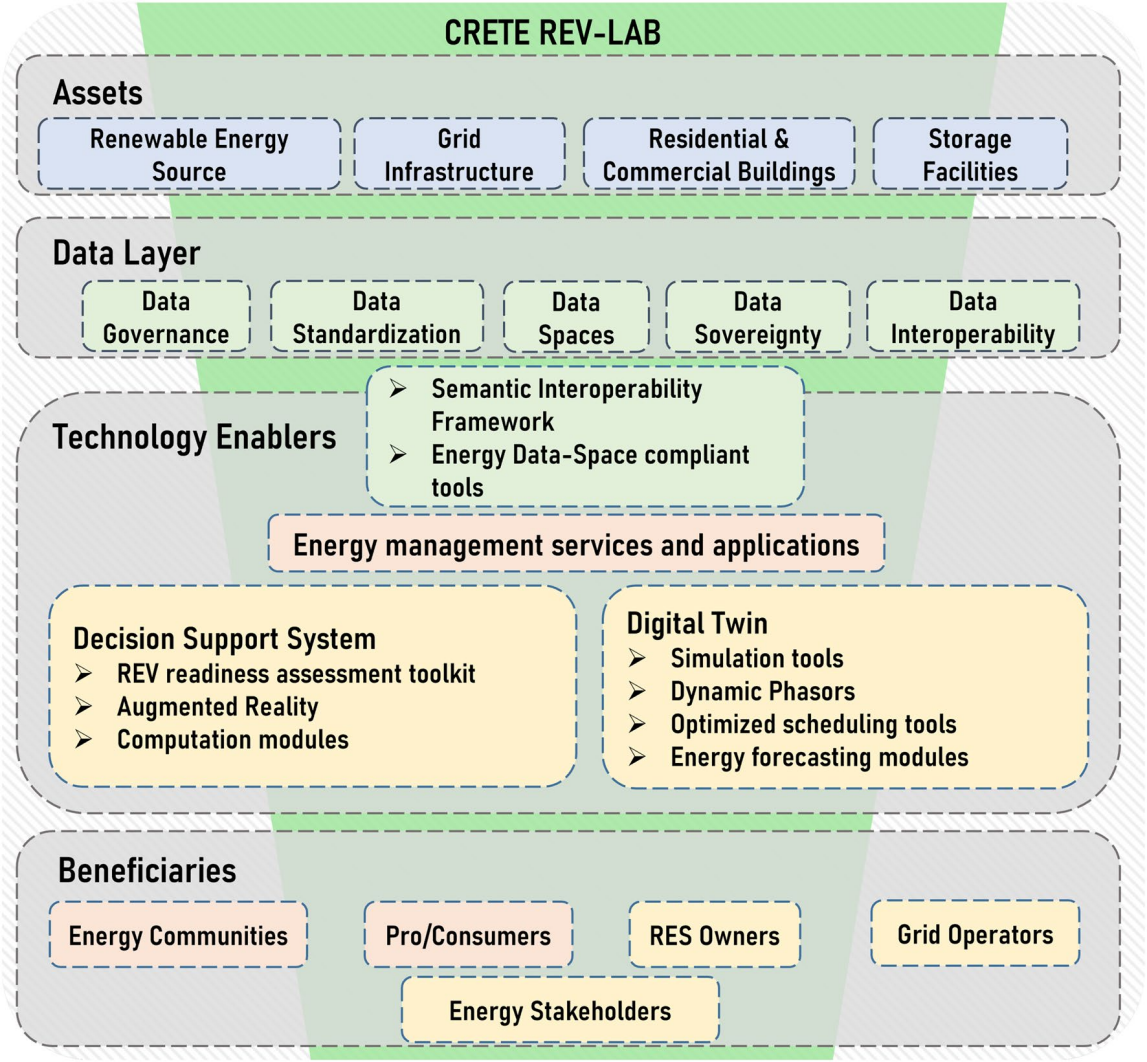
Architecture framework for the digital layer of the proposed CRETE REV-Lab.

The system-of-systems DT will be developed to cater to the needs of various stakeholders within the REV ecosystem. Network operators, including DSOs and TSOs, will utilize the network DT to simulate and optimize grid operations, including the management of network parameters such as line congestion and transformer load. RES owners and producers will have access to individual DTs for each RES within their portfolio, enabling them to monitor and optimize the performance of their assets. Consumers within the energy community in Crete will benefit from energy management services and applications designed to enhance their participation in the energy transition, facilitating the consumption of renewable energy and the management of energy demand in alignment with community goals. A schematic representation depicting all the interdependencies among the deployed technologies in the context of the CRETE REV-Lab is shown in Fig. 3.

### Business models for up-scaling the Crete REV

Lastly, the business layer will provide a comprehensive business sandbox inside the environment of the REV in Crete, facilitating the development and implementation of innovative business models tailored to the unique needs and opportunities of energy communities. This sandbox will incorporate models, roles, and practices for DRES-based systems, emphasizing the integration of energy communities to optimize the exploitation and management of flexibility available at the district level. Specifically, we will study the perception of different stakeholders on Crete REV-Lab, focusing on generating local jobs, skills, economic growth, and benefits for citizens. Through a comprehensive empirical analysis of citizens’ decision preferences and underlying cognitive, affective, and social factors in the context of Crete REV-Lab, we will generate indicators and inspiring practices related to potential job creation, entrepreneurship, local acceptance, circularity, energy performance, and more.

From an economic and technological perspective, state-of-the-art mechanisms will be offered for clustering and segmenting consumers based on multiple criteria such as socio-economic, gender, socio-cultural, and socio-political factors. This segmentation will enable better understanding of behavioral patterns and preferences, leading to the creation of individualized incentive mechanisms that enhance citizen engagement in renewable energy design, implementation, and exploitation. Additionally, we will provide decision support and conflict resolution tools to enhance consensus among stakeholders, facilitated by a user-friendly REV decision support tool that considers sustainability aspects.

Societal benefits of energy communities will be emphasized, including advancing energy efficiency, lowering electricity bills, creating local job opportunities, and attracting private investments in clean energy projects. The Crete REV-Lab is poised to become a dynamic area for investment and employment, leading to new jobs in related sectors such as construction and manufacturing. Furthermore, we recognize the importance of clarifying the concept and implementation of energy communities, ensuring alignment with sustainable community development and energy democracy goals. The goal of the Crete Rev-Lab positions energy communities as locally and collectively organized energy systems, encompassing a range of concepts including sustainable energy communities^56^, community energy, community microgrids^57^, community-based virtual power plants^58^, and prosumer-community groups^59^. These arrangements prioritize the participation of residential and non-residential members who collaborate to reach common goals, with democratic and shared ownership and organization rules^13^. The overarching aim is to fulfill the energy needs of local communities, promoting energy autonomy while delivering social and environmental benefits.

## Discussion and limitations

The proposed REV initiative on the island of Crete pioneers a holistic approach to energy transition, integrating cutting-edge ICT technologies with social innovation processes. By leveraging the island’s diverse geographical entities and climate conditions, the REV-Lab develops tailored solutions utilizing multiple renewable energy carriers. The establishment of CELs as Innovation Hubs enables real-life testing of grid management strategies, ensuring sustainable and cost-effective energy production and storage to meet local needs. While each CEL may have unique characteristics and challenges, the principles and methodologies developed within the Crete REV-Lab provide valuable guidance and inspiration for fostering energy transition and community empowerment initiatives globally. The Crete REV-Lab will be established as a hub of innovative solutions creating valuable knowledge on the green, smart and sustainable transformation of the modern energy landscape. Regions with unique geographical complexities will be able to draw upon best practices of the Crete REV-Lab to utilize accessible RES and achieve energy autonomy. Lessons learned from the Crete REV-Lab will pave the way towards refined engagement methods for citizens. By sharing best practices, lessons learned, and innovative solutions -both in societal and technological aspects- through targeted workshops hosted by the Crete REV-Lab, the knowledge generated from the Crete REV-Lab will contribute towards accelerating the adoption of sustainable energy systems and promoting energy democracy in diverse geographical contexts.

Concurrently, similar ongoing projects worldwide underscore the global momentum towards clean energy transition. However, beyond technological advancements, the success of such initiatives hinges on the cultivation of sustainable communities and their pivotal role in driving ecological transition. The Valley Clean Energy^60^, operational for 5 years, provides power to citizens across California, drawing from 378 MW of solar, hydro, and geothermal power, complemented by energy storage systems. Currently, Valley Clean Energy offers three levels of electricity service tailored to customer needs. Another notable energy valley serving California and neighboring states is Silicon Valley Clean Energy^61^. SVCE boasts over 600 MW of installed capacity from solar, wind, and geothermal energy sources, supported by over 100 MW of utility-scale energy storage. In Europe, the integration of clean energy production and consumption is exemplified by Hydrogen Valley projects. Project HEAVENN^62^, spanning six locations across the Northern Netherlands, aims to optimize the integration of RES such as solar, onshore, and offshore wind power, utilizing hydrogen as a storage medium. Initiated in 2020, HEAVENN aims to become a key energy vector for further decarbonization, contributing to industrial, heating, and transportation sectors. Similarly, the BalticSeaH2 Project^63^ targets a large-scale valley around the Baltic Sea, centered between southern Finland and Estonia and it envisages producing up to 100,000 tonnes of hydrogen annually within 5 years. At the same time, the North Adriatic Hydrogen Valley (NAHV) project’s objective is the creation of a hydrogen-based economic, social and industrial ecosystem based on the capacity of the quadruple helix actors^64^. Additional Hydrogen Valley initiatives include Green Hysland in Mallorca^65^ and BIG HIT in Scotland^66^.

While these global initiatives share the objective of transitioning to clean energy, the proposed demonstration of a REV in Crete stands out in several key aspects. Crete’s REV is uniquely tailored to the island’s diverse geographic and climatic conditions. Each CEL in Crete addresses specific local challenges and opportunities, showcasing a nuanced approach that aligns with the island’s distinct characteristics. In contrast, some other projects operate across larger regions or focus on specific technologies without the same level of geographic adaptability. Various RES are also integrated into the proposed REV to create a comprehensive and resilient energy portfolio, while existing projects emphasize specific energy sources or technologies, lacking the same breadth of diversity. Moreover, Crete’s REV places a strong emphasis on community engagement and ownership through the involvement of citizens in energy communities.

Regarding strategies for developing sustainable communities, current limitations include the lack of comprehensive strategies tailored to the diverse geographical and socioeconomic contexts within each region, hindering the effective implementation of sustainable community initiatives^56,67^. In the context of the Crete REV-Lab, our approach to developing sustainable communities involves a comprehensive consideration of various factors. Firstly, we recognize the diverse potentials within Crete, encompassing geographical, climatic, and natural resource variations, as well as differing needs across sectors such as buildings, mobility, industry, and industrial parks. Notably, we acknowledge significant disparities between the coastal zones and mountainous areas, as well as between the western and eastern regions of Crete. These variations necessitate tailored strategies for each locale to ensure the effectiveness and sustainability of energy community initiatives.

Moreover, present challenges lie in the limited depth of social science expertise and methodological approaches to actively engage and foster social cohesion among energy communities^68^, impeding the establishment of robust social models for ecological transition^69^. Our efforts extend beyond mere technological solutions to encompass social aspects crucial for fostering sustainable communities as new social models for ecological transition. We aim to deepen social science expertise by implementing methodological approaches that facilitate active societal participation in the establishment of the Crete REV-Lab and CELs. These CELs, strategically located across the Crete Valley, serve as focal points for promoting mutual learning and exchanging best practices among energy communities. By fostering a common understanding and social ties among these communities, we seek to catalyze the transition towards sustainable energy practices and resilient community structures.

Last but not least, current limitations include limited access to tools that assist decision-making for REV planning, which makes it harder to make decisions and share important information among stakeholders^70^. To this end, interactive decision support tools will be provided for REV planning that serve multiple functions. Firstly, they assist communities and citizens in decision-making processes related to renewable energy ventures, while also enhancing energy security and accelerating the green transition in Europe. Secondly, these tools offer valuable insights for value chain energy stakeholders, facilitating investment and business opportunities within communities. Lastly, they serve as an information exchange hub for prosumers, communities, and stakeholders, fostering collaboration and knowledge-sharing essential for sustainable community development.

The limitations of implementing the REV in Crete presented in this article encompass various challenges due to the plethora of all the involved stakeholders and the complexity of the project that could potentially impede progress and success. Fragmented stakeholder engagement can hamper cohesive collaboration and alignment of interests. Limited buy-in and support from stakeholders, including communities, governments, and businesses, could hinder the mobilization of resources and commitment. In addition, inadequate coordination mechanisms can result in disjointed efforts and the inefficient use of resources. Finally, regulatory and policy barriers can create uncertainty and obstacles to navigating legal frameworks and obtaining necessary approvals for the construction of the various RES. Addressing these limitations requires proactive efforts to foster collaboration, secure resources and navigate regulatory landscapes.

## Conclusions

In conclusion, our study presents actionable insights for stakeholders considering investments in local or regional renewable energy systems. The Crete REV-Lab advocates for the utilization of a decision support tool to assist investors in strategically selecting the most efficient cluster of renewable energy generation assets, flexible consumers, and DRES. Furthermore, consumers can benefit from participating in electricity markets with clean energy flows, facilitated by a user-operator ‘alliance’ model. This model mitigates renewable energy variability through the use of distributed flexibility from DR, active control, and distributed storage.

The exploration of new business models for flexibility, including P2P local markets and various marketplaces, offers opportunities to enhance revenue streams and encourage broader participation in renewable energy initiatives. Moreover, our emphasis on promoting new business models and market simulation tools underscores the importance of innovation in addressing the challenges of renewable energy integration.

In essence, our study not only highlights the obstacles to reshaping the energy landscape but also provides practical insights into operationalizing REVs. Through the integration of technological innovation (DTs, AI, data spaces), market mechanisms, and community engagement, our proposed strategies pave the way for realizing the full potential of renewable energy resources in driving socio-economic and environmental transformation.

## Data Availability

The datasets used and/or analysed during the current study are available from the corresponding author on reasonable request.

## References

[1] Commission, E. & for Communication, D.-G. *REPowerEU Actions* (Publications Office of the European Union, 2022).

[2] Commission, E. & for Communication, D.-G. *What is the European Green Deal?* (Publications Office, 2019).

[3] Digitalising the energy sector - eu action plan. https://ec.europa.eu/info/law/better-regulation/have-your-say/initiatives/13141-digitalising-the-energy-sector-eu-action-plan (2022). [Online; accessed 29 November 2023].

[4] Commission E (2021). Directorate-General for Employment.

[5] Cf O (2015). Transforming our world: the 2030 agenda for sustainable development.

[6] Butt OM, Zulqarnain M, Butt TM (2021). Recent advancement in smart grid technology: Future prospects in the electrical power network. Ain Shams Eng. J..

[7] D’Adamo I, Mammetti M, Ottaviani D, Ozturk I (2023). Photovoltaic systems and sustainable communities: New social models for ecological transition. the impact of incentive policies in profitability analyses. Renew. Energy.

[8] Parra-Domínguez J, Sánchez E, Ordóñez Á (2023). The prosumer: A systematic review of the new paradigm in energy and sustainable development. Sustainability.

[9] Caramizaru, A., Uihlein, A. *et al.**Energy communities: an overview of energy and social innovation*, vol. 30083 (Publications Office of the European Union Luxembourg, 2020).

[10] Caferra R, Colasante A, D’Adamo I, Morone A, Morone P (2023). Interacting locally, acting globally: Trust and proximity in social networks for the development of energy communities. Sci. Rep..

[11] Lennon B, Dunphy N (2024). Sustaining energetic communities: Energy citizenship and participation in an age of upheaval and transition. Sci. Rep..

[12] Heldeweg MA, Saintier S (2020). Renewable energy communities as ‘socio-legal institutions’: A normative frame for energy decentralization?. Renew. Sustain. Energy Rev..

[13] Reis IF, Gonçalves I, Lopes MA, Antunes CH (2021). Business models for energy communities: A review of key issues and trends. Renew. Sustain. Energy Rev..

[14] Wainer A, Petrovics D, van der Grijp N (2022). The grid access of energy communities a comparison of power grid governance in France and Germany. Energy Policy.

[15] Angelidis, O., Ioannou, A., Friedrich, D., Thomson, A. & Falcone, G. District heating and cooling networks with decentralised energy substations: Opportunities and barriers for holistic energy system decarbonisation. *Energy* 126740 (2023).

[16] Wang Y (2019). Optimal design of integrated energy system considering economics, autonomy and carbon emissions. J. Clean. Prod..

[17] Ilias L, Sarmas E, Marinakis V, Askounis D, Doukas H (2023). Unsupervised domain adaptation methods for photovoltaic power forecasting. Appl. Soft Comput..

[18] Sarmas E, Forouli A, Marinakis V, Doukas H (2024). Baseline energy modeling for improved measurement and verification through the use of ensemble artificial intelligence models. Inf. Sci..

[19] Chabrol M (2016). Re-examining historical energy transitions and urban systems in Europe. Energy Res. Soc. Sci..

[20] Ahlqvist V, Holmberg P, Tangerås T (2022). A survey comparing centralized and decentralized electricity markets. Energ. Strat. Rev..

[21] Abrishambaf O, Lezama F, Faria P, Vale Z (2019). Towards transactive energy systems: An analysis on current trends. Energ. Strat. Rev..

[22] Ullah S, Haidar AM, Hoole P, Zen H, Ahfock T (2020). The current state of distributed renewable generation, challenges of interconnection and opportunities for energy conversion based dc microgrids. J. Clean. Prod..

[23] Zou H (2019). A survey of energy management in interconnected multi-microgrids. IEEE Access.

[24] Alam MN, Chakrabarti S, Ghosh A (2018). Networked microgrids: State-of-the-art and future perspectives. IEEE Trans. Industr. Inf..

[25] Wahlund M, Palm J (2022). The role of energy democracy and energy citizenship for participatory energy transitions: A comprehensive review. Energy Res. Soc. Sci..

[26] Kotilainen, K. *Energy prosumers’ role in the sustainable energy system*, 1–14 (Springer, 2019).

[27] Leal-Arcas, R., Alemany Rios, J. & Akondo, N. Energy decentralization in the European union. *Georgetown Environ. Law Rev.* **32** (2019).

[28] Markov KK, Rajaković N (2019). Multi-energy microgrids with ecotourism purposes: The impact of the power market and the connection line. Energy Convers. Manage..

[29] Michalakopoulos V (2024). A machine learning-based framework for clustering residential electricity load profiles to enhance demand response programs. Appl. Energy.

[30] Testasecca, T., Lazzaro, M., Sarmas, E. & Stamatopoulos, S. Recent advances on data-driven services for smart energy systems optimization and pro-active management. In *2023 IEEE International Workshop on Metrology for Living Environment (MetroLivEnv)*, 146–151 (IEEE, 2023).

[31] Shen H, Liu Y, Xia Z, Zhang M (2020). An efficient aggregation scheme resisting on malicious data mining attacks for smart grid. Inf. Sci..

[32] Kallio L, Heiskanen E, Apajalahti E-L, Matschoss K (2020). Farm power: How a new business model impacts the energy transition in Finland. Energy Res. Soc. Sci..

[33] Wilkinson S, Hojckova K, Eon C, Morrison GM, Sandén B (2020). Is peer-to-peer electricity trading empowering users? Evidence on motivations and roles in a prosumer business model trial in australia. Energy Res. Soc. Sci..

[34] Botelho D, de Oliveira L, Dias B, Soares TA, Moraes C (2022). Prosumer integration into the Brazilian energy sector: An overview of innovative business models and regulatory challenges. Energy Policy.

[35] Commission welcomes deal on electricity market reform https://www.pubaffairsbruxelles.eu/eu-institution-news/commission-welcomes-deal-on-electricity-market-reform/. [Online; accessed 8 March 2024].

[36] Norbu S, Couraud B, Robu V, Andoni M, Flynn D (2021). Modelling the redistribution of benefits from joint investments in community energy projects. Appl. Energy.

[37] Gjorgievski VZ, Cundeva S, Georghiou GE (2021). Social arrangements, technical designs and impacts of energy communities: A review. Renew. Energy.

[38] Campos I, Marín-González E (2020). People in transitions: Energy citizenship, prosumerism and social movements in europe. Energy Res. Soc. Sci..

[39] Pan, H. et al. Digital twin and its application in power system. In *2020 5th International Conference on Power and Renewable Energy (ICPRE)*, 21–26 (IEEE, 2020).

[40] Onile AE, Machlev R, Petlenkov E, Levron Y, Belikov J (2021). Uses of the digital twins concept for energy services, intelligent recommendation systems, and demand side management: A review. Energy Rep..

[41] Palensky P, Cvetkovic M, Gusain D, Joseph A (2022). Digital twins and their use in future power systems. Digital Twin.

[42] Arraño-Vargas F, Konstantinou G (2022). Modular design and real-time simulators toward power system digital twins implementation. IEEE Trans. Industr. Inf..

[43] Saad, A., Faddel, S. & Mohammed, O. Iot-based digital twin for energy cyber-physical systems: Design and implementation. *Energies* **13**. 10.3390/en13184762 (2020).

[44] Brown D, Kivimaa P, Sorrell S (2019). An energy leap? business model innovation and intermediation in the ‘energies prong’retrofit initiative. Energy Res. Soc. Sci..

[45] Brassar, M. et al. Regulatory aspects of self-consumption and energy communities. the council of european energy regulators report (2019).

[46] Brinker L, Satchwell AJ (2020). A comparative review of municipal energy business models in Germany, California, and Great Britain: Institutional context and forms of energy decentralization. Renew. Sustain. Energy Rev..

[47] Sarmas E, Spiliotis E, Stamatopoulos E, Marinakis V, Doukas H (2023). Short-term photovoltaic power forecasting using meta-learning and numerical weather prediction independent long short-term memory models. Renew. Energy.

[48] Mirz M, Vogel S, Reinke G, Monti A (2019). Dpsim-a dynamic phasor real-time simulator for power systems. SoftwareX.

[49] Holttinen H (2012). Methodologies to determine operating reserves due to increased wind power. IEEE Trans. Sustain. Energy.

[50] Vourdoubas, J. Use of renewable energy sources for energy generation in rural areas in the island of crete, greece. *Eur. J. Environ. Earth Sci.* **1** (2020).

[51] Katsaprakakis, D. A. *et al.* Greek islands’ energy transition: From lighthouse projects to the emergence of energy communities. *Energies* **15**, 10.3390/en15165996 (2022).

[52] Wang H, Jiang K, Shahidehpour M, He B (2019). Reduced-order state space model for dynamic phasors in active distribution networks. IEEE Trans. Smart Grid.

[53] Sarmas E, Dimitropoulos N, Marinakis V, Mylona Z, Doukas H (2022). Transfer learning strategies for solar power forecasting under data scarcity. Sci. Rep..

[54] Chorozoglou, S., Sarmas, E. & Marinakis, V. An integrated ml-ops framework for automating ai-based photovoltaic forecasting. In *2023 IEEE International Conference on Big Data (BigData)*, 3921–3928 (IEEE, 2023).

[55] Sarmas E (2022). An incremental learning framework for photovoltaic production and load forecasting in energy microgrids. Electronics.

[56] Koirala BP, Koliou E, Friege J, Hakvoort RA, Herder PM (2016). Energetic communities for community energy: A review of key issues and trends shaping integrated community energy systems. Renew. Sustain. Energy Rev..

[57] Rathnayaka AD, Potdar VM, Dillon TS, Kuruppu S (2015). Formation of virtual community groups to manage prosumers in smart grids. Int. J. Grid Util. Comput..

[58] Eklund M, Khalilpour K, Voinov A, Hossain M (2023). Understanding the community in community microgrids: A conceptual framework for better decision-making. Energy Res. Soc. Sci..

[59] Rathnayaka AD, Potdar VM, Dillon T, Hussain O, Kuruppu S (2014). Goal-oriented prosumer community groups for the smart grid. IEEE Technol. Soc. Mag..

[60] Valley clean. energy. https://valleycleanenergy.org. [Online; accessed 29 November 2023].

[61] Silicon valley clean energy. https://svcleanenergy.org. [Online; accessed 29 November 2023].

[62] Heavenn. https://heavenn.org. [Online; accessed 29 November 2023].

[63] Balticseah2. https://balticseah2valley.eu. [Online; accessed 29 November 2023].

[64] Nahv. https://www.nahv.eu/. [Online; accessed 29 November 2023].

[65] Greenhysland. https://greenhysland.eu. [Online; accessed 29 November 2023].

[66] Bighit. https://www.bighit.eu. [Online; accessed 29 November 2023].

[67] Santos E, Carvalho M, Martins S (2023). Sustainable water management: Understanding the socioeconomic and cultural dimensions. Sustainability.

[68] Pillan M, Costa F, Caiola V (2023). How could people and communities contribute to the energy transition? conceptual maps to inform, orient, and inspire design actions and education. Sustainability.

[69] Sovacool BK (2023). Social innovation supports inclusive and accelerated energy transitions with appropriate governance. Commun. Earth Environ..

[70] Standal K (2023). Can renewable energy communities enable a just energy transition? Exploring alignment between stakeholder motivations and needs and eu policy in Latvia, Norway, Portugal and Spain. Energy Res. Soc. Sci..

